# Habitat Loss Through Renewable Energy Infrastructure: Budgeting the Energetic Consequences for Wintering Geese

**DOI:** 10.1007/s00267-026-02632-9

**Published:** 2026-09-26

**Authors:** Lisa Vergin, Jesper Madsen, Hans Linssen, Bart A. Nolet, Kevin Kuhlmann Clausen

**Affiliations:** 1 Department of Ecoscience, Aarhus University, Aarhus, Denmark; 2 Department of Theoretical and Computational Ecology, Institute for Biodiversity and Ecosystem Dynamics, University of Amsterdam, Amsterdam, The Netherlands; 3 Department of Animal Ecology, Netherlands Institute of Ecology, Wageningen, The Netherlands

**Keywords:** Taiga Bean Goose, green transition, scenario simulations, body mass dynamics, energy budget, winter severity

## Abstract

The global expansion of renewable energy is a key societal objective but may conflict with nature conservation interests by causing habitat loss. Hence, planning energy infrastructure requires a thorough understanding of the behavior and habitat requirements of affected species during critical phases of the annual cycle. We assessed the energetic consequences of losing agricultural foraging habitat for a wintering goose species with a high degree of site-fidelity. Proposed renewable energy developments, specifically wind turbines and solar panels, pose a risk of losing winter cereal fields, which are the prime foraging habitat for the geese during cold spells. Using estimates of energy intake and expenditure derived from GPS-tagged individuals, we simulated body mass development under cold conditions with and without access to winter cereals as well as under mild conditions with pastures only. Across all cold winter scenarios, simulated body mass decreased, reflecting lower energy intake as well as increased thermoregulatory costs, and energetic deficits intensified when winter cereals were unavailable. Under mild winter conditions, body mass could be maintained without access to winter cereals. These results demonstrate that habitat loss has considerable energetic consequences for wintering geese under cold winter conditions. More broadly, our findings underscore the need to assess land-use change in the context of both resource availability and environmental conditions. Ensuring access to high-quality foraging habitats during energetically challenging conditions should therefore be prioritized in spatial planning. Where habitat loss is unavoidable, alternative habitats must provide comparable resource quality, low disturbance, and behavioral compatibility, particularly for site-faithful species.

## Introduction

Renewable energy infrastructure is expanding rapidly and altering landscapes worldwide. While essential for addressing the effects of climate change (European Environment Agency 2023), these energy developments can conflict with nature conservation and affect biodiversity by modifying, fragmenting or destroying habitats (Gasparatos et al. 2017; Gorman et al. 2023). This conflict is amplified by the fact that energy projects are often located away from human settlements, thereby frequently overlapping with areas of nature conservation interests (Svensson et al. 2023). As habitat degradation is a well-recognized threat to bird populations (Kirby et al. 2008), expanding energy infrastructure may pose additional pressures on populations. Habitat and landscape destruction may influence resident species in the immediate vicinity of the developments but also affect migratory species that depend on a memorized network of suitable habitats across their annual cycle (Newton 2008; Xu et al. 2019; Beal et al. 2025).

Migratory waterfowl, and geese in particular, are often highly site-faithful to both non-breeding and breeding sites (Fox et al. 2002; Phillips et al. 2003; Thornton et al. 2021), which can make them especially vulnerable to changes in landscape and habitat composition. Geese use several habitats throughout the annual cycle to meet distinct functional requirements and varying energetic demands, affecting energy accumulation, migration, and, ultimately, reproduction and survival (Cadieux et al. 2005; Kéry et al. 2006; Klaassen et al. 2006; Davis et al. 2014; Lameris et al. 2021). During the wintering period, individuals focus on survival and aim to maintain their body condition (e.g., Lameris et al. 2021; Owen et al. 1992). The loss of suitable habitats during this phase can negatively affect body condition and potentially cause carry-over effects to subsequent stages of the annual cycle (Clausen et al. 2015; Fowler et al. 2020). Thus, quantifying the energetic consequences of changes in winter foraging site availability, such as habitat loss caused by infrastructure development, is essential for guiding effective conservation measures on the wintering grounds.

Wintering site selection in migratory geese is shaped by multiple factors such as food quality and availability, landscape structure, perceived predation risk (including disturbance by humans), and distance to night roosts (Madsen 1998; Béchet et al. 2004; Clausen et al. 2012; Rosin et al. 2012; Chudzińska et al. 2015; Jensen et al. 2017; Pot et al. 2019). These environmental factors influence the energetic budget, defined by the difference between energy intake and energy expenditure (e.g., Clausen et al. 2012). Cold winter conditions lead to elevated thermoregulatory costs, which consequently increase energy expenditure and, with that, the need for higher energy intake (Lameris et al. 2021; Vergin et al. 2025). Furthermore, frost and snow can limit access to food resources, potentially reducing foraging efficiency and thus lowering energy intake (Vergin et al. 2025). Over the past decades, agricultural intensification in northern Europe has created new foraging opportunities for geese (Van Eerden et al. 2005), enabling them to more easily meet their energetic needs during periods of increased thermoregulatory demand and to maintain a suitable energy budget throughout winter (Fox and Abraham 2017). Agricultural fields provide high-energy food that can support greater energy intake compared to natural habitats (Madsen 1985), making them a frequently used resource during winter (Fox and Abraham 2017; Vergin et al. 2025).

Recent plans for renewable energy developments pose a risk of losing agricultural foraging habitats used by wintering Taiga Bean Geese (*Anser fabalis fabalis*) in Denmark (Clausen and Madsen 2023). An energy cluster, consisting of wind turbines, solar panels, and biogas plants, is proposed within the species’ traditional cold-weather refuge area, specifically on winter cereal fields that are intensively used during cold spells (periods of sub-zero weather conditions during winter) (Vergin et al. 2025). The Taiga Bean Goose is a species of conservation concern, reflecting past population declines, and the protection of its key habitats is considered essential (Marjakangas et al. 2015). Moreover, their strong site fidelity (Thornton et al. 2021) suggests a limited capacity to exploit new sites and compensate for habitat loss. Yet, the energy cluster installations may increase collision risks for the geese and may cause disturbances and displacements, potentially preventing the geese from exploiting the traditionally used winter cereal fields (Drewitt and Langston 2006; Clausen and Madsen 2023). Understanding the energetic consequences of losing these winter cereal fields is critical, as it remains unclear how such habitat changes may affect the energy budget and ultimately winter survival of Taiga Bean Geese, particularly during cold spells.

In this study, we aim to assess the energetic consequences of agricultural habitat loss due to the development of an energy cluster for wintering Taiga Bean Geese. We modeled body mass development during several different scenarios with or without winter cereals. We simulated a worst-case scenario in which winter cereals were unavailable throughout a prolonged cold spell, restricting the geese to forage exclusively on semi-natural pastures (pasture-cold scenario), and contrasted this with parallel scenarios in which winter cereal availability was maintained, and used either fully (winter cereal-cold scenario) or in combination with semi-natural pastures (combined habitat use-cold scenario). To further contrast the worst-case scenario (pasture-cold) with a mild winter scenario, we simulated body mass development under mild conditions with no use of winter cereal habitat (pasture-mild scenario). By combining habitat-specific foraging conditions with weather-dependent energetic modeling, this study illustrates an approach for evaluating the consequences of land-use change for migratory waterfowl.

## Methods

## Study Area and Focal Population

**Fig. 1:**
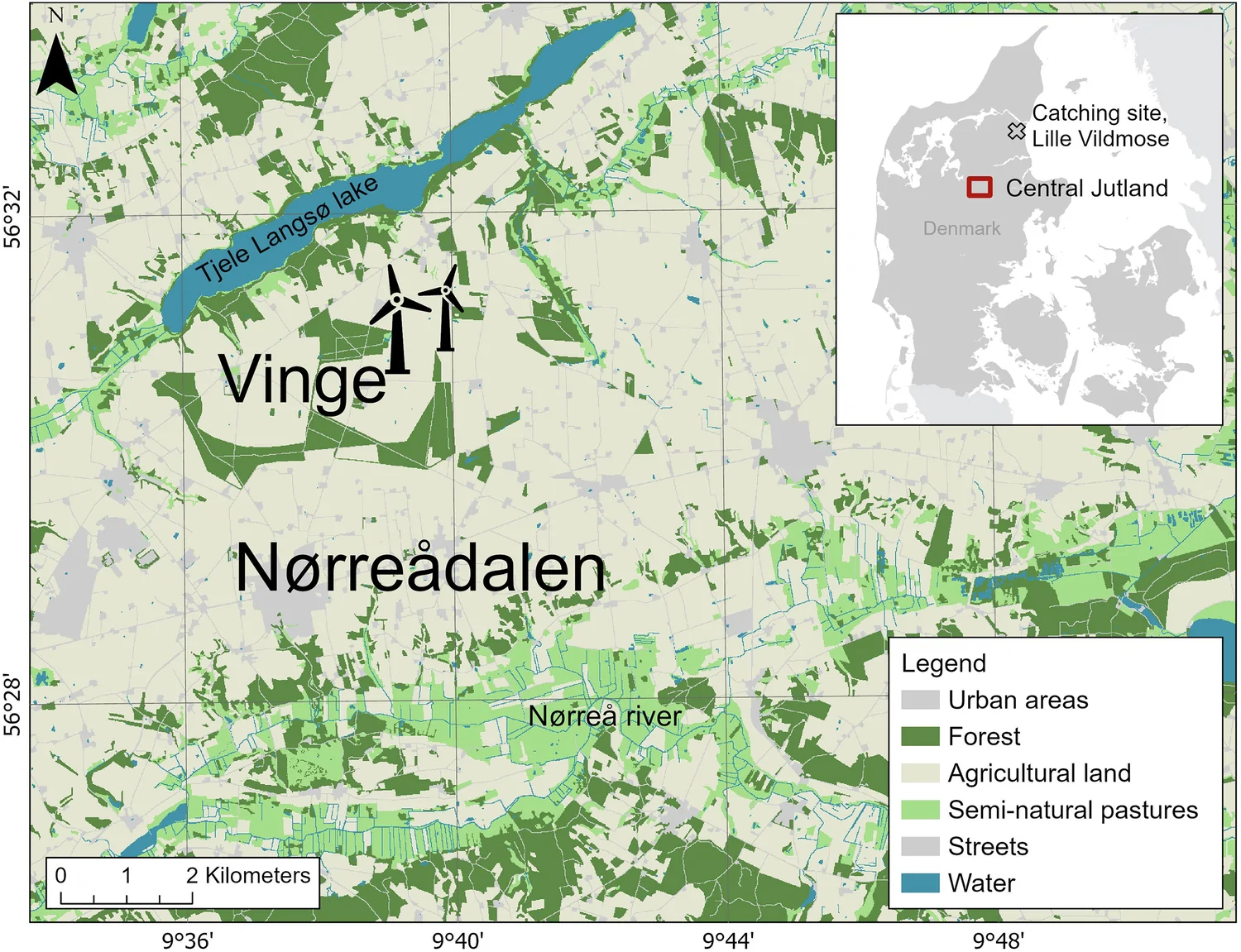
Study area showing the wintering sites Vinge and Nørreådalen used by Taiga Bean Geese in central Jutland, Denmark. Vinge is characterized by agricultural fields, especially winter cereals, and is the focus area of a planned renewable energy cluster. Lake Tjele Langsø is the nearest night roost used by geese in this region. Nørreådalen is characterized by wet semi‑natural pastures, with night roost possibilities on the Nørreå river or on nearby (permanently or temporarily) flooded pastures. GPS‑tracked individuals included in the analysis were captured in Lille Vildmose, approximately 60 km northeast of these wintering sites. The map was created in ArcGIS Pro v3.1.0

In central Jutland, Denmark, wintering Taiga Bean Geese rely on both agricultural fields in Vinge (56°31′ N, 9°39′ E) as well as on semi-natural pastures in the adjacent river valley Nørreådalen (56°27′ N, 9°41′ E; Fig. 1) (Vergin et al. 2025). Night roosts were located within approximately 2 km of foraging sites in both Vinge and Nørreådalen. In Nørreådalen, geese roost on a river or on flooded pastures, whereas in Vinge they roost on a lake. Nørreådalen has previously been identified as core area for wintering Taiga Bean Geese in central Jutland (Brandt et al. 2017; Clausen et al. 2024), and semi-natural pastures as the predominantly used habitat (Vergin et al. 2025). The intensive use of Nørreådalen is likely supported by its location in a natural, less disturbed landscape (cf. Clausen et al. 2013). In Vinge, Taiga Bean Geese appear more susceptible to disturbance, which frequently causes them to leave the area for the remainder of the day (Vergin et al. 2025). However, during cold spells, the green winter cereals in Vinge become particularly important and intensively used, as they provide a high‑energy food resource, reflecting the temperature-related habitat use of Taiga Bean Geese (Vergin et al. 2025).

Taiga Bean Geese wintering in central Jutland belong to a subgroup of the population breeding in northeastern Norway, northern Sweden, Finland, and northwest Russia, and number approximately 1500–2000 individuals (Marjakangas et al. 2015; Clausen et al. 2024; Sørensen et al. 2025). Their main wintering site, and first site used after arrival from Sweden, is Lille Vildmose in the northeastern part of Denmark (Fig. 1). However, nearly all geese of this subgroup, including the geese represented in this study, move from Lille Vildmose to Vinge in central Jutland during cold winters (Brandt et al. 2017; Clausen et al. 2024), highlighting the role of this area as a key cold-weather refuge that enables the geese to respond to cold spells (Vergin et al. 2026).

The agricultural fields in Vinge are the focus of a planned renewable energy cluster (Clausen and Madsen 2023). Suitable alternative winter cereal fields close to the main night roosts appear limited, and GPS-tracked geese have shown to return to the same winter cereal fields across years (Vergin et al. in prep), underscoring their high importance. Currently, a designated protective area has been established to preserve the habitats for geese in Vinge (Clausen and Madsen 2023). However, under the full development of the energy cluster, wind turbines and solar panels would overlap with the foraging fields of Taiga Bean Geese, which is expected to result in the complete loss of the current foraging habitat through direct habitat loss caused by solar panels and displacement associated with wind turbines (e.g., Rees et al. 2012). As a compensatory measure, the establishment of winter cereal fields on existing cultivated land in Nørreådalen has been proposed within the project area to provide foraging habitat should the fields in Vinge become unavailable (Clausen et al. 2024).

## **GPS-tagging**

In November 2023, Taiga Bean Geese were captured in Lille Vildmose in northeastern Jutland, Denmark, using remote-controlled clap nets with domesticated live decoys (Dutch Association of Goose Catchers). Eleven adult individuals were fitted with solar-powered GPS-GSM neck collars (OrniTrack N44, Ornitela), of which seven (six females, one male) used the area in central Jutland during the same wintering season (winter 2023/2024). Depending on battery levels, GPS fixes were obtained at intervals of 10 to 60 min, and triaxial accelerometer data were collected concurrently for 2 s at 20 Hz.

## Body Mass Development Under Different Energetic Scenarios

To examine the energetic consequences of a potential complete loss of the winter cereal fields in Vinge associated with the expansion of an energy cluster, we modeled body mass development with or without winter cereals. Particular emphasis was placed on cold winter scenarios, as these represent the critical conditions under which winter cereals become an essential food resource for Taiga Bean Geese. During mild conditions, the geese forage almost exclusively on pastures in Nørreådalen, and, consequently, cold spells provide the most relevant context for evaluating the energetic consequences of winter cereal loss. Given the current intensive use of Nørreådalen by the geese and plans by the renewable energy project to provide alternative winter cereal fields there, we treated this area as the core wintering area in our simulations. Accordingly, in scenarios where winter cereals were available, we assumed that alternative winter cereal fields had been established on cultivated land within Nørreådalen. This allowed us to simulate both semi-natural pasture and winter cereal foraging within a single area. By doing so, we minimized variation in local movements and thereby focused the comparison on differences in habitat availability and winter severity. The worst-case of our scenarios restricted geese to (1) foraging exclusively on semi-natural pastures during a prolonged cold spell (pasture-cold scenario), thereby mimicking the complete loss of the agricultural habitat associated with the proposed energy cluster. This was contrasted with scenarios in which winter cereals remained available under the same cold winter conditions. For these alternative scenarios, we simulated body mass development for geese (2) foraging exclusively on winter cereals (winter-cereal-cold scenario) as well as (3) foraging in combination with semi-natural pastures (combined-habitat-cold scenario). For this combined scenario, we assumed that foraging time was split evenly between winter cereals and semi-natural pastures, which reflects the proportional use of these habitats seen in prolonged cold periods (Vergin et al. 2025). To compare the worst-case scenario with a prolonged mild winter, we simulated body mass development in (4) a hypothetical mild winter when geese forage exclusively on semi-natural pastures (pasture-mild scenario).

We simulated body mass development of Taiga Bean Geese under different scenarios by adapting the framework of Lameris et al. (2021), who calculated daily energy budget and subsequent changes in body mass from daily energy intake and expenditure for Barnacle Geese (*Branta leucopsis*). Here, we applied this structure to Taiga Bean Geese by using species-specific values wherever available. The simulations of our study were based on average population values, and GPS-tracking data were used to parameterize the scenarios with time spent on the foraging grounds and behavior-specific energy costs (see details below). The GPS-tracking dataset was derived from Taiga Bean Geese wintering in central Jutland during 2023/2024, where weather- and habitat-specific energetic measurements of food and dropping samples had previously been collected (Vergin et al. 2025). For this winter, daily locations of GPS-tracked geese were available between 4 January and 28 February, and this period was used to simulate body mass development. We used mean November catching weights of the GPS-tracked geese visiting central Jutland as initial body mass in all scenarios (mean body mass = 3487 ± 142 g SE), as no data on January body mass of Taiga Bean Geese in Denmark were available. The observed capture masses fall within the range reported for both males and females (Kirwan et al. 2024). However, given the small and female-biased sample (six of seven individuals), our data do not allow sex-specific estimates of behavior or body mass development. The simulations should therefore be interpreted as representing the average population scenario reflected by this sample. Although the initial body mass determines the absolute values of body mass-dependent parameters, and consequently the absolute body mass development of a scenario, the main focus of this study lies on relative differences between scenarios rather than on absolute values.

To ensure consistency between the energetic measurements and the simulated weather conditions, the hypothetical cold and mild winter scenarios were parameterized using weather data from the same season. Plant and fecal samples representing cold conditions were collected on days with a daily average temperature below 0 °C, associated with snow and frost, whereas samples representing mild conditions were collected on days with temperatures above 0 °C (Vergin et al. 2025). The same temperature threshold was therefore used to define the simulated weather scenarios. Hence, cold winter scenarios were parameterized using the mean weather conditions recorded on days with a daily average temperature below 0 °C during the winter 2023/2024, and the mild scenario was parameterized using the corresponding mean values from days with a daily average temperature above 0 °C (Supplementary Table S1). These values were held constant throughout each simulation, representing prolonged periods of persistently cold or mild weather. While day-to-day variation in weather would influence absolute body mass development, it would also affect the scenarios equally. Consequently, it would have no impact on comparisons among scenarios within the same weather treatment, as these were simulated under the same environmental conditions.

### Energy Expenditure

Daily energy expenditure was calculated from basal metabolic rate (BMR), energetic costs associated with specific behaviors (activity costs), and thermoregulatory costs to maintain body temperature under sub-thermoneutral conditions. As no direct BMR measurements are available for Taiga Bean Geese, we used reference measurements from a closely related species with a similar body mass, the Greylag Goose (*Anser anser*), to scale BMR values to Taiga Bean Geese of varying body mass (in the scenarios), following the linear approximation over a narrow mass range described by Lameris et al. (2021):

$${{BMR}}_{i}\,=\,\frac{{W}_{i}}{{W}_{{ref}}}\,\times {BM}{R}_{{ref}}$$

with *W*_*i*_ being the body mass of a Taiga Bean Goose at a certain point in time, *W*_*ref*_ = 3229 g and *BMR*_*ref*_ = 9.02 W (Greylag Goose; Bruinzeel et al. 1997).

#### Behavior Classifications and Activity Costs

To quantify activity costs, we developed a behavior classification model based on the tri-axial accelerometer data from tagged Taiga Bean Geese, using the rabc package in R (Yu and Klaassen 2021; R Core Team 2025). Behavioral training data were obtained by filming tagged individuals in the field while simultaneously recording bursts of accelerometer data (2 s, 20 Hz, each burst representing one behavioral sample). Video recordings were annotated using the program BORIS (Friard and Gamba 2016). In total, six individuals were annotated for 6.75 h, comprising 3.17 h on winter cereals and 3.58 h on semi-natural pastures. The model was trained to classify five behavioral categories: foraging (*n* = 4368 samples), preening (*n* = 880), sleeping (*n* = 1144), resting (*n* = 3129), and flying (*n* = 197). Foraging included head-down searching, feeding while standing or walking or lying, and walking in between head-down searches for food. Preening comprised grooming of different feather groups. Sleeping was defined as head tucked under the wing. Resting included inactive behavior when birds were awake. Flying was annotated from active flights recorded during filming. Rare behaviors such as drinking, head-shaking, or transitions between postures were classified as other behavior and excluded from model training. The classification model was fit using the rabc package routine, calculating the package-default set of frequency- and time-domain features, partitioning the samples into a training (70%) and testing (30%) set, and fitting an extreme gradient boosting (XGBoost) model using five-fold cross-validation (Yu and Klaassen 2021). The final model had an overall accuracy of 91% and a Cohen’s Kappa statistic of 0.87 (see Supplementary Table S2 for classification performance statistics per behavioral class and Supplementary Fig. S1 for a confusion matrix).

Behavioral annotation was limited to daytime observations. Consequently, model performance was expected to be higher for daytime behavior than for nocturnal behavior when birds roosted on water and exhibited different movement patterns, such as swimming. While sleeping and inactive states are likely classified reliably at night, other categories may contain misclassifications. For example, foraging was occasionally predicted for birds roosting on lakes, where feeding was not possible. These potential night-time misclassifications may slightly overestimate overall activity costs.

Behavior classifications were used to calculate proportional time allocation per behavior for each tracked individual and day, yielding individual daily activity budgets. To resemble local flight costs within Nørreådalen, activity budgets were parameterized based on GPS-tracked individuals that remained within that area. Behavior-specific energy costs were calculated as the product of the proportional time allocation, BMR and a behavior-specific BMR multiplier: 1.6 for foraging, 1.5 for inactive behavior (sleeping and resting), and 1.9 for preening (Stahl 2001; Lameris et al. 2021). The BMR multiplier for flight was estimated using the allometric relationship of Butler and Bishop (2000) following Ladin et al. (2011), corresponding to 13.7 × BMR for Taiga Bean Geese. The sum of all behavior-specific energy costs over a day represented the total daily activity costs.

Using a BMR based on a fixed winter body mass of 3,487 g, daily activity costs neither varied over time nor differed between GPS-tracked birds that spent the whole day in Nørreådalen, mainly foraging on semi-natural pastures, and those that spent the whole day in Vinge, mainly foraging on green leaves of winter cereals (see Supplementary Section S1). Hence, to simulate semi-natural pasture and winter cereal foraging within the same spatial unit, we calculated a mean value and standard deviation of behavior proportions based on the geese in Nørreådalen across the study period.

#### Thermoregulation and Daily Energy Expenditure

Thermoregulatory costs were estimated as a function of body mass, ambient temperature, wind speed, and global radiation using the functions provided by Baveco et al. (2011) and Lameris et al. (2021), following the model developed by Cartar and Morrison (1997). We assumed a body temperature of 40 °C, an average goose plumage resistance of 867 s m⁻¹, reflecting the plumage insulation (averaged over measurements of Barnacle and Brent Geese (*Branta bernicla*) as no species-specific measurements are available for Taiga Bean Geese; see van der Graaf et al. (2001) and Baveco et al. (2011)), and wind speed adjusted to bird height (0.15 m above ground; Baveco et al. 2011; Lameris et al. 2021). Weather input variables were obtained from the Danish Meteorological Institute (DMI). For the cold scenarios, we used the mean temperature, wind speed and global radiation of days with daily mean temperatures <0 °C during winter 2023/2024; for the mild scenario, corresponding means were calculated from days with daily mean temperatures >0 °C (Supplementary Table S1).

Following Lameris et al. (2021), we assumed that heat produced during activity contributes to thermoregulation, such that additional thermoregulatory costs were only paid when environmental heat loss exceeded activity-derived heat production. For each behavior category, thermoregulatory costs were calculated over the corresponding proportional time allocation, and realized energy expenditure for that allocation was defined as the maximum of behavior-specific energy costs and thermoregulatory costs. Total daily energy expenditure was calculated as the sum of realized energy expenditures per day.

### Energy Intake

Energy intake per habitat and weather condition was estimated from measurements reported by Vergin et al. (2025). Intake was estimated using dropping rates obtained with the hourly block method, combined with ash-free dry mass of feces, acid detergent fiber content (ADF) and gross energy content of both plant material and feces as well as the time spent per habitat (see Supplementary Section S2).

For our calculations, we quantified time spent on foraging grounds during daytime using the GPS tracking data of Taiga Bean Geese in central Jutland. To account for an increase in day length over the season, we fitted a generalized additive model (GAM) with daily time on the foraging grounds as the response variable, day as a smooth predictor, and individual as a random intercept, using the mgcv package in R (Wood 2011; R Core Team 2025). We drew 500 samples from the posterior distribution of the fitted GAM and summarized these to obtain a daily mean and standard deviation. Predicted values from this model were used to parameterize daytime foraging duration in the scenario simulations.

Taiga Bean Geese may supplement daytime energy intake through occasional nocturnal foraging, as has been reported for Barnacle Geese, particularly during moonlit nights (Lameris et al. 2021). To account for seasonal variation associated with the lunar cycle, we again fitted a GAM with nocturnal foraging duration as the response, day as a smooth predictor, and individual as a random intercept (Wood 2011; R Core Team 2025). We drew 500 samples from the posterior distribution and used the predicted values of a daily mean and standard deviation to estimate nocturnal energy intake (see Supplementary Section S3). Foraging duration at night was parameterized using GPS data from individuals roosting in Nørreådalen. To account for potential misclassification of nocturnal behaviors, we only considered foraging behavior assignments with classification probabilities above 90%. In Nørreådalen, GPS-tracked geese roosted on the river or on flooded semi-natural pastures that both allowed feeding in immediate proximity. As no active nocturnal flights to distant feeding fields were detected in our data, energetic measurements from pasture habitats were used to estimate nocturnal intake.

We had no data on nocturnal foraging of geese foraging on winter cereals during the day, as most individuals subsequently roosted on a lake in Vinge with no opportunities for nocturnal foraging. We therefore assumed that nocturnal foraging duration in Nørreådalen was identical across all simulated habitat scenarios and served to supplement daily energy intake in winter, regardless of the foraging habitat during daytime. Consequently, any uncertainty associated with the estimation of nocturnal foraging duration affects all scenarios equally and should not bias comparisons among them. Daily energy intake was then calculated as the sum of daytime and nighttime energy intake.

### Body Mass Development

In order to standardize simulations of body mass development over our study period (4 January to 28 February; 56 days), we used average population parameters (initial body mass of 3487 g; see above) and calculated daily energy budget for each scenario as the difference between daily energy intake and daily energy expenditure. Following Lameris et al. (2021), energy surplus (positive energy budget) was converted to body mass at a rate of 1/29 g/kJ (29 kJ/g energy density of deposited tissue measured for a female Pink-footed Goose (*Anser brachyrhynchus*); Madsen and Klaassen 2006), assumed to be stored with an efficiency of 0.8. Conversely, when daily energy expenditure exceeded intake (negative energy budget), body stores were assumed to be burned, applying the same energy-to-mass conversion, with an efficiency of 1. The resulting body mass was used as the starting body mass for the subsequent day, and body mass-dependent parameters, such as BMR, were recalculated daily from the updated simulated body mass. Consequently, activity and thermoregulatory costs dependent on BMR also changed daily throughout the simulations. Body mass-independent parameters, including energy intake parameters (dropping rates as well as plant and feces specific measurements; Supplementary Table S5) and behavioral time allocation (Supplementary Table S4), remained fixed within each scenario and were therefore unaffected by simulated changes in body mass. Thus, the model does not include behavioral or energetic compensation for body mass loss, such as increased foraging effort.

Since daily energy intake was the only varying model input between cold winter scenarios, differences in simulated body mass development between these can be linked directly to those intake changes. To summarize how body-mass differences accumulated over time and to assess the effect of excluding winter cereal foraging, we calculated the proportional difference in simulated body mass between scenarios for each day and fitted a linear regression. The regression was used to estimate the slope of the linear relationship between proportional body-mass difference and time.

### Uncertainty Analysis

Our simulations represent population-level body mass development and were parameterized using mean values for daily foraging time (both day- and nighttime) and behavior proportions derived from individual GPS data, together with values used to estimate energy intake (dropping rates, ADF, and gross energy values) derived from flock-level measurements. To reflect uncertainty in these measured estimates, we performed 500 stochastic simulations for each scenario. For each day in the simulations, uncertain parameters were sampled prior to calculating daily energy intake and expenditure. GPS-derived foraging time and behavior proportion were randomly sampled from normal distributions defined by the respective mean and standard deviation, reflecting uncertainty in the individual-based estimates. Sampled behavior proportions were truncated to the interval 0 to 1 and subsequently normalized to ensure that daily proportions summed to 1. Parameters derived from flock-level measurements were sampled from normal distribution defined by the respective mean and standard error, reflecting uncertainty in the population estimates. From these simulations, we calculated the daily mean body mass per scenario and the corresponding 95% confidence intervals.

## Results

To assess possible consequences of loss of winter cereal foraging fields caused by energy infrastructure development, we simulated energy budgets and body mass development for wintering Taiga Bean Geese across scenarios differing in winter severity and the availability of winter cereals over a 56-day period.

**Fig. 2:**
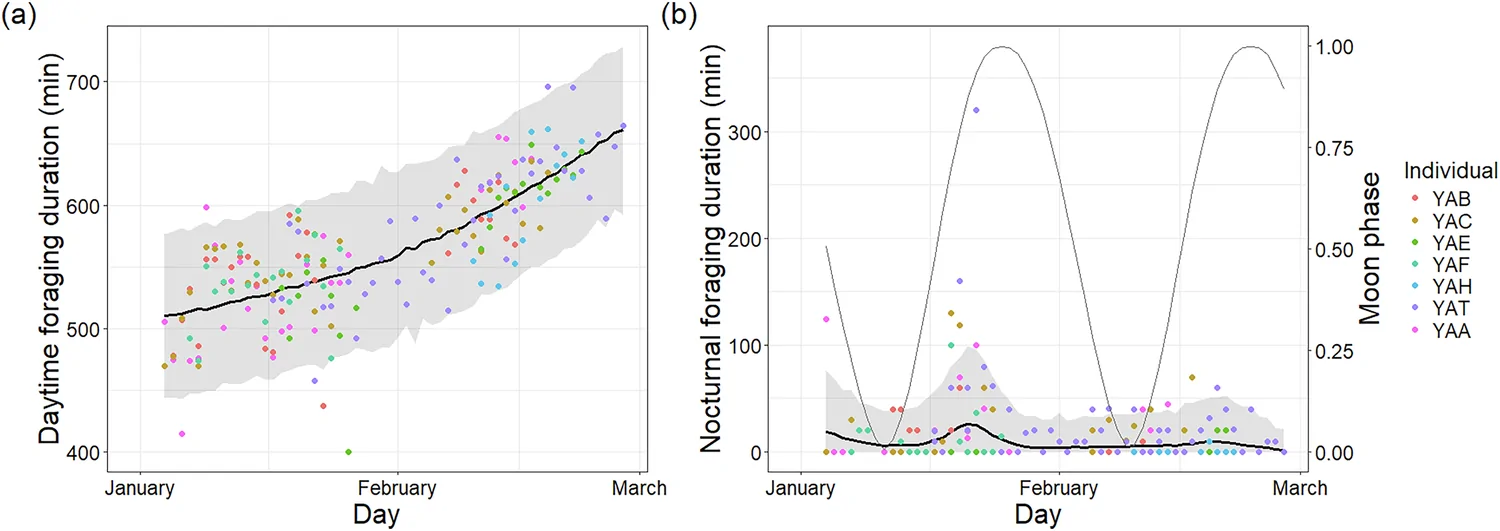
Daily foraging duration of Taiga Bean Geese during (**a**) day- and (**b**) nighttime from 4 January until 28 February. Solid lines represent the predictions from the fitted GAM, with shaded areas representing the 95% confidence intervals based on posterior draws from the corresponding model. Dots represent the raw data; different colors represent different individuals. The sinusoidal line illustrates the lunar cycle across the same period, 0 representing new moon and 1 representing full moon. Note that in (**a**) the y-axis does not start at zero

**Fig. 3:**
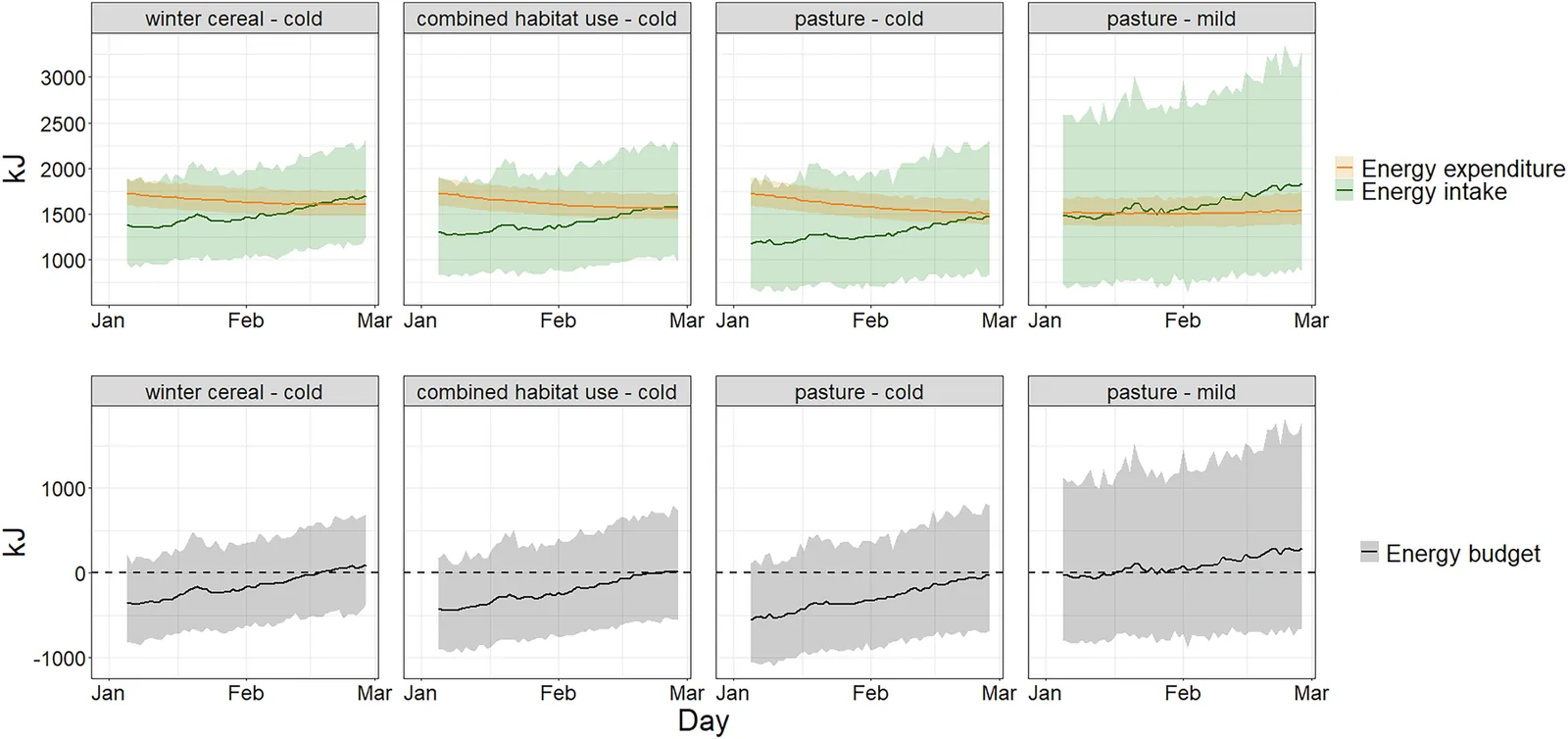
Simulated daily energy intake, energy expenditure, and resulting energy budget for Taiga Bean Geese with an initial body mass of 3487 g under different winter scenarios with contrasting habitat configurations between 4 January and 28 February. Cold winter scenarios represent a prolonged cold spell with constant conditions based on the average weather conditions of days with daily average temperatures <0 °C during the corresponding period in 2024, whereas mild winter scenarios represent constant weather based on days >0 °C. Within the cold scenarios, three habitat configurations were simulated: exclusive daytime foraging on winter cereal fields, exclusive daytime foraging on semi-natural pastures, and combined use of both habitats. For the mild winter scenario, only foraging on semi-natural pastures was simulated, reflecting the predominant use of winter cereals during colder conditions. The solid lines represent the modeled energy intake (green), energy expenditure (orange) and energy budget (black); the shaded areas indicate the 95% confidence interval. The geese are in energy balance at kJ = 0 in the lower panels (dashed line)

Following the winter solstice, increasing day length was associated with a gradual extension of time spent on foraging grounds by GPS-tracked Taiga Bean Geese between January and February, reaching approximately 30% higher values by the end of February (Fig. 2a). Nocturnal foraging varied over the season, with increased activity during periods of higher lunar illumination (Fig. 2b). Together, longer duration on the foraging grounds and elevated nocturnal foraging contributed to higher overall energy intake. Daily energy intake varied between 1167 and 1835 kJ across all scenarios. In cold scenarios, intake was highest in simulations including only foraging on winter cereals (winter cereal-cold; range: 1349–1698 kJ), intermediate for combined habitat use of winter cereals and pastures (combined habitat use-cold; range: 1268–1582 kJ), and lowest when no winter cereals and only pastures were available (pasture-cold; range: 1167–1482 kJ). In the pasture-mild scenario, intake varied between 1443 and 1835 kJ (Fig. 3).

The behavior proportions used to estimate activity-based expenditure are presented in Table 1. Based on an initial body mass of 3487 g, energy expenditure varied between 1,496 and 1728 kJ across scenarios. Under cold winter conditions, expenditure was elevated due to increased thermoregulatory costs (winter cereal-cold: 1602–1728 kJ; combined habitat use-cold: 1559–1727 kJ; pasture-cold: 1503–1726 kJ), whereas under mild conditions it primarily reflected activity-related costs (pasture-mild: 1496–1543 kJ) (Fig. 3). The magnitude of differences between scenarios changed over time, reflecting the scenario-specific developments in body mass.

**Table 1:** Daily behavior proportions (mean and standard deviation, SD) for wintering Taiga Bean Geese, which were used to estimate daily behavior-specific energy expenditure

Behavior	Mean proportion	SD
Foraging	0.41	0.09
Preening	0.10	0.06
Resting	0.44	0.11
Sleeping	0.03	0.03
Flying	0.01	0.01

**Fig. 4:**
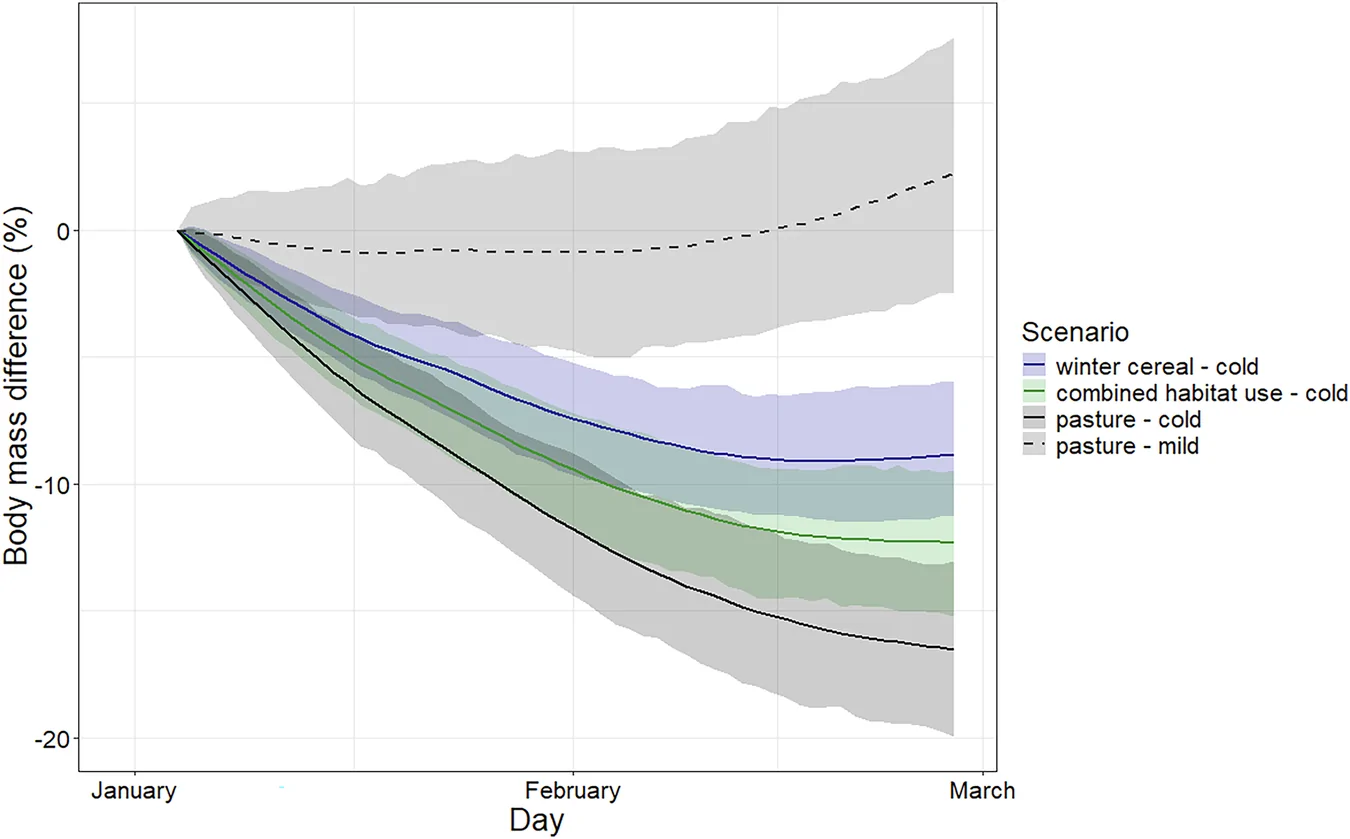
Simulated relative body mass development shown as the relative difference for Taiga Bean Geese starting with an initial body mass of 3487 g between 4 January and 28 February under differing winter and habitat scenarios (described in the caption of Fig. 3). Solid lines represent the modeled body mass development under cold scenarios; the dashed line represents the mild scenario; the shaded areas indicate the 95% confidence intervals

The combined effects of intake and expenditure determined the simulated energy budget across scenarios. Across all cold-winter scenarios, elevated energy expenditure, together with reduced energy intake under cold conditions, generally resulted in negative energy budgets and an overall decline in simulated body mass (Fig. 3). This effect was, however, most pronounced in the pasture-cold scenario, where body mass decreased more strongly than in simulations including winter cereal availability. By the end of the simulation, body mass in the pasture-cold scenario was up to 8% lower than in scenarios with access to winter cereals (difference to winter cereal-cold: –7.75%, 95% CI: (–11.82, –3.36); difference to combined habitat use-cold: –4.15%, 95% CI: (–8.59, 0.44); Fig. 4).

Under cold-winter conditions, differences in habitat-specific energy intake caused the proportional difference in body mass between scenarios to increase almost linearly over time (*R*² = 0.996). The proportional difference in body mass between the pasture-cold and winter cereal-cold scenario increased by 0.14% per day, while the proportional difference between the pasture-cold and combined habitat use-cold scenario increased by 0.08% per day. In our scenarios, this corresponded to an additional body mass loss of –4.86 ± 0.04 g/day for geese restricted to semi-natural pastures compared to exclusively foraging on winter cereals during daytime, and to –2.67 ± 0.02 g/day compared to mixed foraging on winter cereals and semi-natural pastures. These differences accumulated progressively over time, such that the duration of the cold spell increased the divergence in body mass between scenarios and amplified the negative effect of lacking access to winter cereals. Simulated absolute body mass development is shown in Supplementary Fig. S2.

During a prolonged period of constant mild winter conditions, energy budget remained neutral when geese foraged exclusively on the semi-natural pastures (pasture-mild). Consequently, simulated body mass stayed more or less stable across the simulated period (Figs. 3,  4).

## Discussion

Changes in habitat availability can have considerable energetic consequences for wintering waterfowl (Clausen et al. 2012). Our study shows that access to energetically profitable habitats plays an important buffering role during periods of elevated thermoregulatory demands. The simulations of Taiga Bean Geese revealed that habitat loss associated with renewable energy construction may cause reduction of body mass over time, if winter cereal habitat becomes unavailable during cold winter conditions. The effect was most pronounced when contrasting scenarios assuming exclusive foraging on winter cereals with those assuming exclusive use of semi-natural pastures. In simulations assuming combined use of both habitats, which represents a more typical habitat utilization observed for Taiga Bean Geese during cold periods, body mass declined as well, though the effect was less distinct. Moreover, the magnitude of the negative effect increased with the duration of a cold spell, implying a correspondingly increasing need for geese to compensate to restore body stores. Under the constant mild‑winter scenario, body mass was maintained throughout the study period while foraging exclusively on semi‑natural pastures. In a hypothetical scenario of exclusive winter cereal foraging in mild winters, the higher energy intake on this crop (Vergin et al. 2025) would, however, predict an unrealistic increase in body mass (Supplementary Fig. S3), given that geese generally aim to maintain body mass throughout winter (e.g., Lameris et al. 2021). This reflects a structural limitation of the model, which does not include feedback from energetic state on foraging behavior (e.g., duration, intake rate, habitat choice), thereby preventing behavioral adjustments that would, in reality, regulate body mass. That pasture habitats may be sufficient to sustain energy balance under mild winter conditions aligns with previous work showing that Taiga Bean Geese can meet their energetic requirements with less energy-rich food when thermoregulatory demands remain low (Vergin et al. 2025).

The simulated cold scenarios in our study represent severe winter conditions. Under winters with shorter cold spells and intermittent milder periods, cumulative body mass loss would likely be lower. Simulated total body mass reductions of 9-17% in our cold winter scenarios are highly sensitive to the assumption of constant weather conditions, and absolute body mass developments will differ under more variable weather conditions. Likewise, absolute body mass development also depends on the initial body mass. However, our focus was on the relative differences between scenarios. As environmentally varying conditions and different initial body mass would affect all scenarios equally, these limitations are unlikely to have biased our results. Our simulations further assume that alternative winter cereal fields are located within Nørreådalen. While this assumption is appropriate for our case study, greater travel distances to alternative winter cereal fields would increase flight costs by 10.5 J/m (Kölzsch et al. 2023) and thus overall energy expenditure. Despite these limitations, the body mass reductions predicted in our model are broadly comparable to declines reported in other studies of wintering geese. For instance, Clausen et al. (2012) modeled a decline of approximately 20% in wintering Brent Geese over a two-month period, and Owen et al. (1992) estimated reductions of up to 13% across a winter in Barnacle Geese. Clausen et al. (2015) reported an 8% difference in body mass of Pink-footed Geese between cold and mild winters, based on abdominal profile indexes converted to body mass following Madsen and Klaassen (2006).

Despite the general warming trend in winter temperatures due to climate change, anticipated increases in climatic variability (Thornton et al. 2014; Xu et al. 2020) imply that cold spells during winter are likely to persist across years, suggesting that geese will continue to rely on the availability of cold‑weather foraging habitats in future winters. Recent weather records likewise illustrate the continued occurrence of cold spells during winter. In Denmark, over the past 16 years, 12 winters included at least one continuous cold spell with daily mean temperatures below zero lasting a week or longer (range: 7-33 days). During winter 2026, January and February together included 44 days with subzero temperatures (two cold spells separated by one week with temperatures above zero; Supplementary Fig. S4), approaching the duration of the prolonged cold conditions represented in our simulations. In our model, the cold scenarios assumed a cold spell with constant weather conditions. This assumption simplified natural weather dynamics, as real cold spells typically include fluctuating weather conditions. Chill factors such as lower temperatures or stronger winds further increase heat loss and thereby elevate thermoregulatory costs (van der Graaf et al. 2001). Within the framework of our cold-winter scenarios, however, such variability would affect thermoregulatory costs equally across all habitat configurations, and the relative differences in body mass between scenarios are not expected to be substantially altered.

Body stores accumulated along the migratory route are essential for fueling migratory flights, subsequent breeding as well as survival (Owen and Black 1989; Schmutz and Ely 1999; Fox et al. 2003; Spaans et al. 2007). Winter severity can have direct consequences for survival in waterfowl, as harsh conditions elevate energetic demands that may lead to declines in body condition, which can in turn reduce survival probability (Bergan and Smith 1993; Clausen et al. 1998; Robb et al. 2001; Nicolai et al. 2026). Furthermore, body condition at the end of winter is important, as it determines how individuals enter spring migration and influences their progression through subsequent stages, with individuals in better condition generally better prepared for the remainder of the migratory cycle (Guillemain et al. 2008). Moreover, higher body condition of female geese at stopover sites prior to arrival at the breeding grounds has been associated with higher reproductive success (Ebbinge and Spaans 1995; Drent et al. 2003; Spaans et al. 2007; Klaassen et al. 2017). Together, these relationships highlight that winter conditions can generate carry-over effects across stages of the annual cycle, such that reduced body condition may ultimately contribute to reduced fitness (e.g., Clausen et al. 2012).

Geese may compensate for reduced body mass by increasing their overall foraging effort, for instance, by extending their nocturnal foraging duration. During winter, daylight hours restrict the potential for additional daytime activity, and previous work suggests that the duration of daytime foraging offers only limited scope for further extension, while increased nocturnal foraging has been shown to support higher winter body mass in wintering Barnacle Geese (Lameris et al. 2021). In our scenarios, we assumed the same level of nocturnal foraging across all scenarios. During prolonged cold spells, geese may spend more time foraging at night to further increase their daily energy intake, especially in scenarios without winter cereals, where energy deficits become more pronounced. However, the extent of this response likely depends on prevailing night-time temperatures and on the balance between the energetic gains from additional foraging and the conservation of energy, and further expansion of nocturnal foraging may also be restricted, as nocturnal activity on terrestrial habitat exposes geese to increased predation risk and is limited by the availability of suitable moonlight (Tinkler et al. 2009; Lameris et al. 2021).

Another potential compensatory strategy could involve exploiting alternative high-energy food resources. In Nørreådalen, Taiga Bean Geese also forage on agriculturally improved grass fields, although the use of these fields showed no detectable weather-related pattern and reflected only moderate use (Vergin et al. 2025). We were, however, unable to simulate scenarios that included foraging on agricultural grass fields in Nørreådalen, as no energetic measurements from plant and dropping samples from this habitat were available to estimate energy intake. Agricultural grass generally has a higher energy content than semi-natural pastures (cf. Lameris et al. 2021) and its use could potentially reduce the difference in body mass development between scenarios with and without winter cereals. Still, winter cereals provide even higher energy content (cf. Therkildsen and Madsen 2000), and it is unlikely that foraging on agricultural grass during prolonged cold conditions alone would fully compensate for the loss of winter cereals.

Compensation could also occur beyond the wintering grounds. For example, Clausen et al. (2015) reported that Pink-footed Geese were able to compensate for reduced body conditions caused by harsh winter conditions along the migratory route, thereby avoiding carry-over effects to the breeding grounds. Energy-rich foraging habitats, such as winter cereals, constitute an important foraging resource for wintering Pink-footed Geese, particularly during cold weather (Therkildsen and Madsen 2000). If these fields would have been unavailable, harsh winters would likely have had an even greater negative impact, and subsequent compensation might have required greater effort. Compensation at staging sites further requires the continued availability of suitable foraging habitats, without further habitat degradation along the migratory route. Overall, geese might be able to (partially) compensate for the absence of winter cereals with different strategies, but the magnitude and effectiveness of such compensation remain speculative. Ensuring continued access to high-energy foraging resources may therefore be the most reliable way to avoid potential negative energetic consequences during winter.

The transition towards renewable energy is an essential component of efforts to mitigate climate change (European Environment Agency 2023). For example, the Danish Climate Act of 2020 sets the target of substantially reducing greenhouse gas emissions and achieving climate neutrality by 2050, with the expansion of renewable energy forming a key component of this strategy (Mollgaard et al. 2022). To avoid negative consequences for geese arising from habitat loss associated with such developments, one approach can be to provide suitable alternative foraging habitats in compensatory areas. Implementing such measures effectively, however, requires a thorough understanding of the habitat characteristics that make alternative fields suitable and attractive, as well as of the behavioral characteristics of the relevant species that influence field selection.

To determine what makes a foraging field suitable for migratory geese, it is important to examine fine-scale field selection and the factors influencing these choices beyond habitat type alone. For wintering Taiga Bean Geese, previous studies showed selection of large fields located farther from public roads and fields with higher elevation (Rosin et al. 2012; Thornton et al. 2021). The latter represents a prominent feature of the winter cereal fields used by geese in Vinge. Additional landscape characteristics may also be important, including the distance to suitable night roosts or proximity to woodland or hedgerows. In addition to such landscape attributes, field use may also be strongly influenced by behavioral factors, including individual experience, the presence of conspecifics, and site fidelity (Fox et al. 2002; Phillips et al. 2003; Rosin et al. 2012), highlighting the potential importance of tradition and memory in shaping foraging site selection. In Taiga Bean Geese, site fidelity appears to be especially pronounced (Thornton et al. 2021). Approximately 70% of all Taiga Bean Geese tagged in Denmark repeatedly used central Jutland as a cold-weather refuge across multiple winters, and use within central Jutland was further concentrated in the same core areas across years (Vergin et al. in prep). For goose species that exhibit a high degree of site-fidelity, the most effective approach for establishing alternative high-energy foraging habitats may be to convert the land use of fields already familiar to the geese. Due to the uncertainties related to field selection, and the potential for altering habitat availability locally, such measures could be implemented within an adaptive management framework by establishing alternative foraging fields before any energy infrastructure is constructed and by testing whether geese keep using these fields. In this way, geese could learn and adjust to alternative habitats before the loss of currently used foraging sites. In our scenarios, we assumed that alternative winter cereal fields were already present within Nørreådalen, although their precise location was not specified. This allowed us to isolate the effect of habitat availability while keeping local flight movements equal across scenarios. Locating alternative winter cereal fields close to the relatively undisturbed natural areas in Nørreådalen may provide conditions like those experienced when geese forage on semi-natural pastures.

## Conclusions

Cold winter conditions represent energetically demanding periods for geese. Our simulations indicate that these challenges become even greater when high-energy foraging habitats are unavailable, particularly when cold conditions persist over extended periods. These findings highlight that maintaining access to high-energy food resources during periods of elevated energetic demand can be critical for sustaining body mass development in wintering geese. As land use continues to change, balancing the expansion of renewable energy infrastructure with the conservation of key foraging habitats used during critical phases of the annual cycle should be an important consideration in future management planning. Designation of alternative sites within goose wintering areas should consider low levels of disturbance, familiarity, and high-quality forage accessibility as essential factors.

## Supplementary information

02 r2 revision supplementary material

## Data Availability

The data and scripts used for the analyses are available from the corresponding author on reasonable request.
