# Supplementary material for: Habitat Loss Through Renewable Energy Infrastructure: Budgeting the Energetic Consequences for Wintering Geese
Source: Environ Manage. 2026 Sep 26;76(10):331. doi: 10.1007/s00267-026-02632-9 (PMC13615994; doi:10.1007/s00267-026-02632-9)
Supplement: Supplementary file 1 [file 267_2026_2632_MOESM1_ESM.pdf]

Supplementary Information

In relation to: Vergin L*, Madsen J, Linssen H, Nolet BA, Clausen KK. Habitat loss through renewable energy infrastructure: budgeting the energetic consequences for wintering geese.

*corresponding author (lve@ecos.au.dk), Department of Ecoscience, Aarhus University, C.F. Møllers Allé 8, 8000 Aarhus C, Denmark

Table S1 Average weather variables used in the constant cold and mild weather scenarios

	Cold winter scenario	Mild winter scenario
Temperature [°C]	-2.57	4.41
Radiation [W/m²]	16.18	27.37
Windspeed at birds' height [m/s]	0.82	1.18

Table S2 Classification performance statistics per behavior class

	Sensitivity	Specificity	Pos Pred Value	Neg Pred Value	Precision	Recall	Prevalence	Detection Rate	Detection Prevalence	Balanced Accuracy
Flying	0.949	0.999	0.933	0.999	0.933	0.949	0.020	0.019	0.020	0.974
Foraging	0.960	0.921	0.904	0.967	0.904	0.960	0.439	0.421	0.466	0.940
Other	0.000	1.000	NA	0.977	NA	0.000	0.023	0.000	0.000	0.500
Preening	0.663	0.983	0.792	0.968	0.792	0.663	0.088	0.059	0.074	0.823
Sleeping	0.971	0.999	0.991	0.996	0.991	0.971	0.115	0.112	0.113	0.985
Resting	0.955	0.960	0.917	0.979	0.917	0.955	0.314	0.300	0.327	0.958

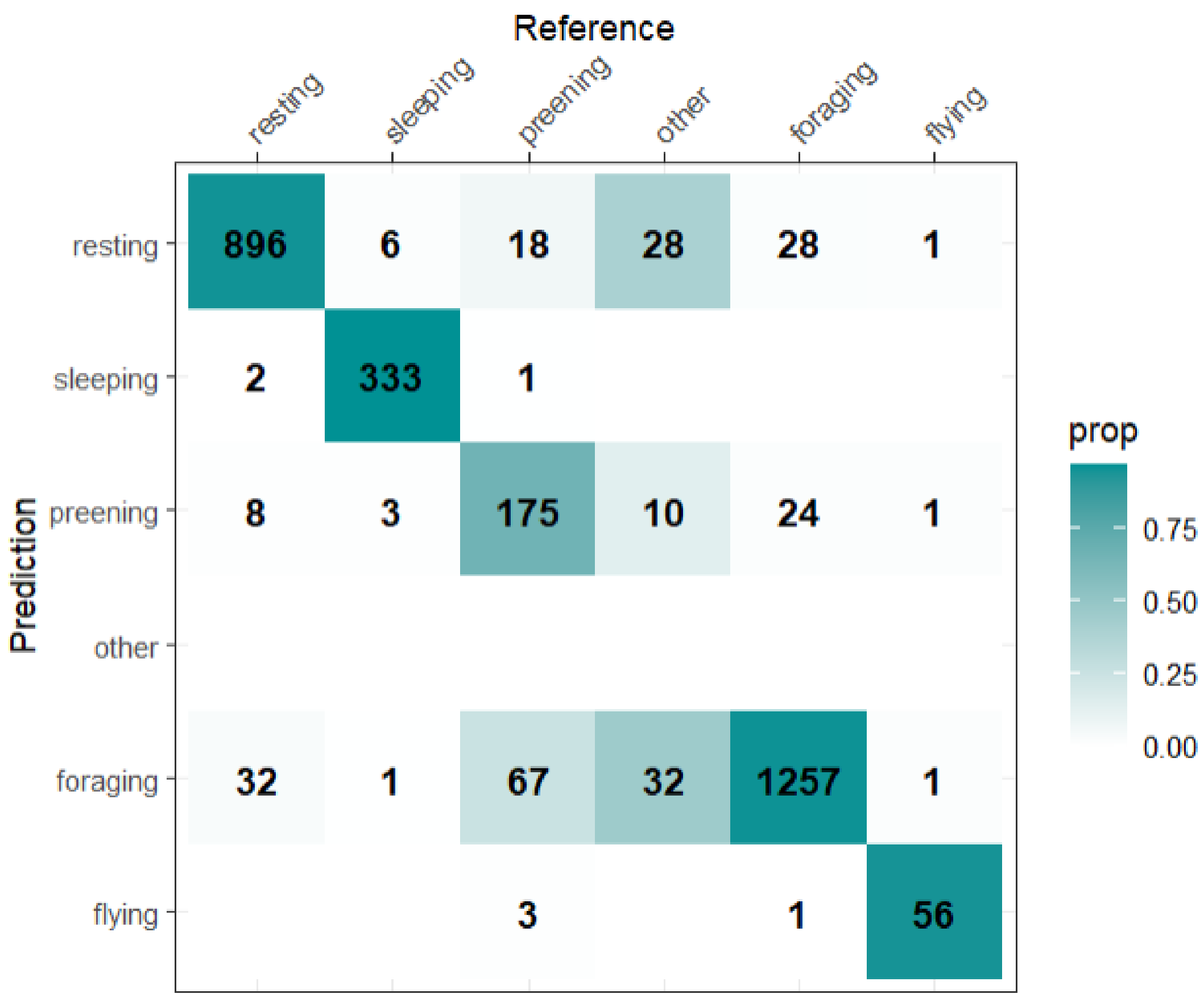

Fig. S1 Confusion matrix of the behavior classification model. Model performance was evaluated using a test dataset comprising 30% of the annotated data, with the remaining 70% used for training. Rows represent model predictions and columns represent reference (annotated) behaviors. Cell values indicate classification frequencies for each predicted–observed combination. Darker shading denotes higher counts, with correct classifications occurring along the diagonal. The behavior category "other" was included in the annotations to capture infrequent behaviors but was excluded from the set of classes predicted by the model

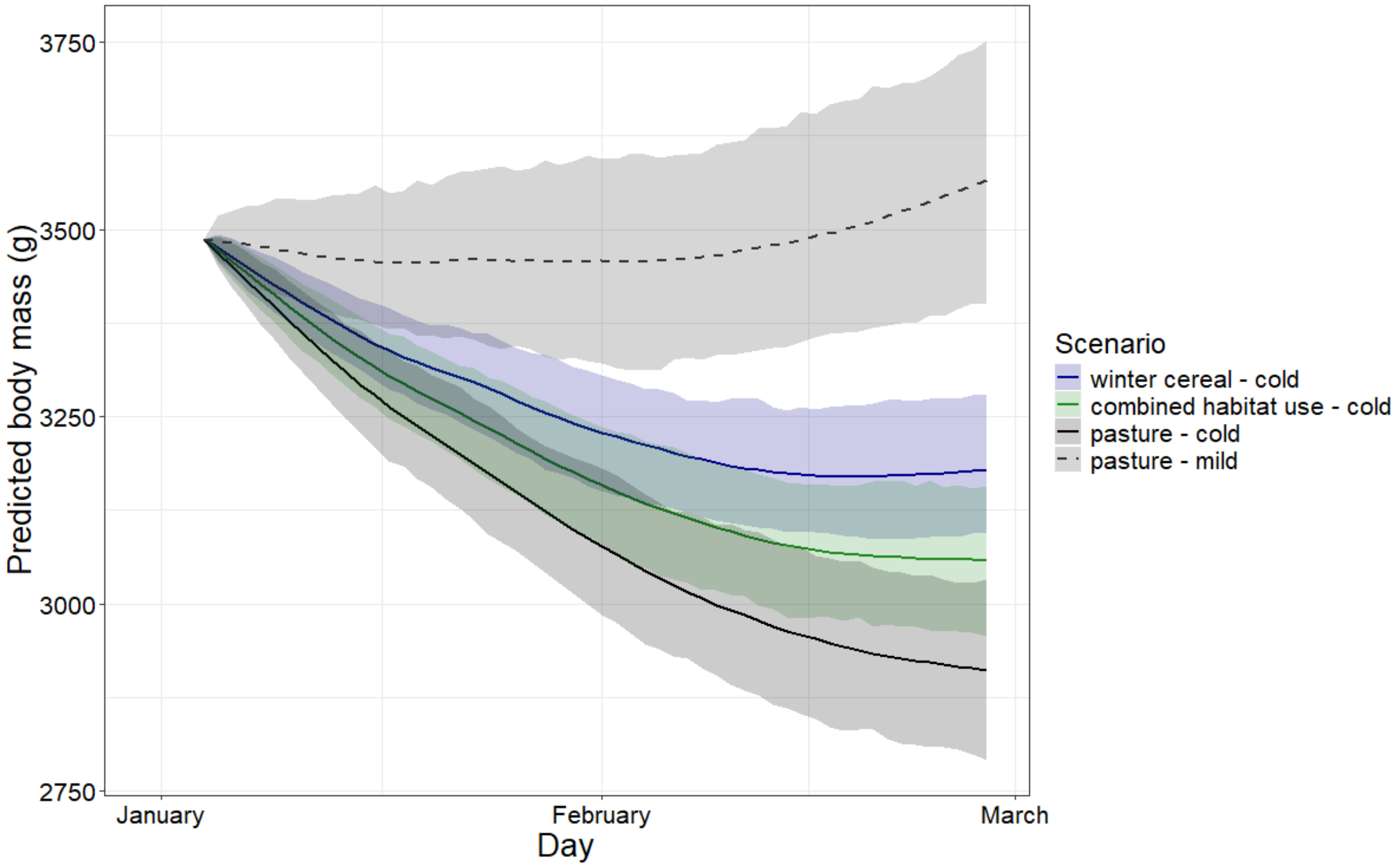

Fig. S2 Absolute body mass development for Taiga Bean Geese starting from an initial body mass of 3,487 g between 4 January and 28 February. The initial body mass was calculated based on capture weights collected in November. Body mass was simulated under constant daily weather conditions (see Table S1) and across different foraging habitats. The absolute body mass development shown here is specific to the assumptions of our simulations and would differ under more variable weather conditions or another initial body mass. Solid lines represent the modelled body mass development under cold scenarios; the dashed line represents the mild scenario; the shaded areas indicate the 95% confidence intervals

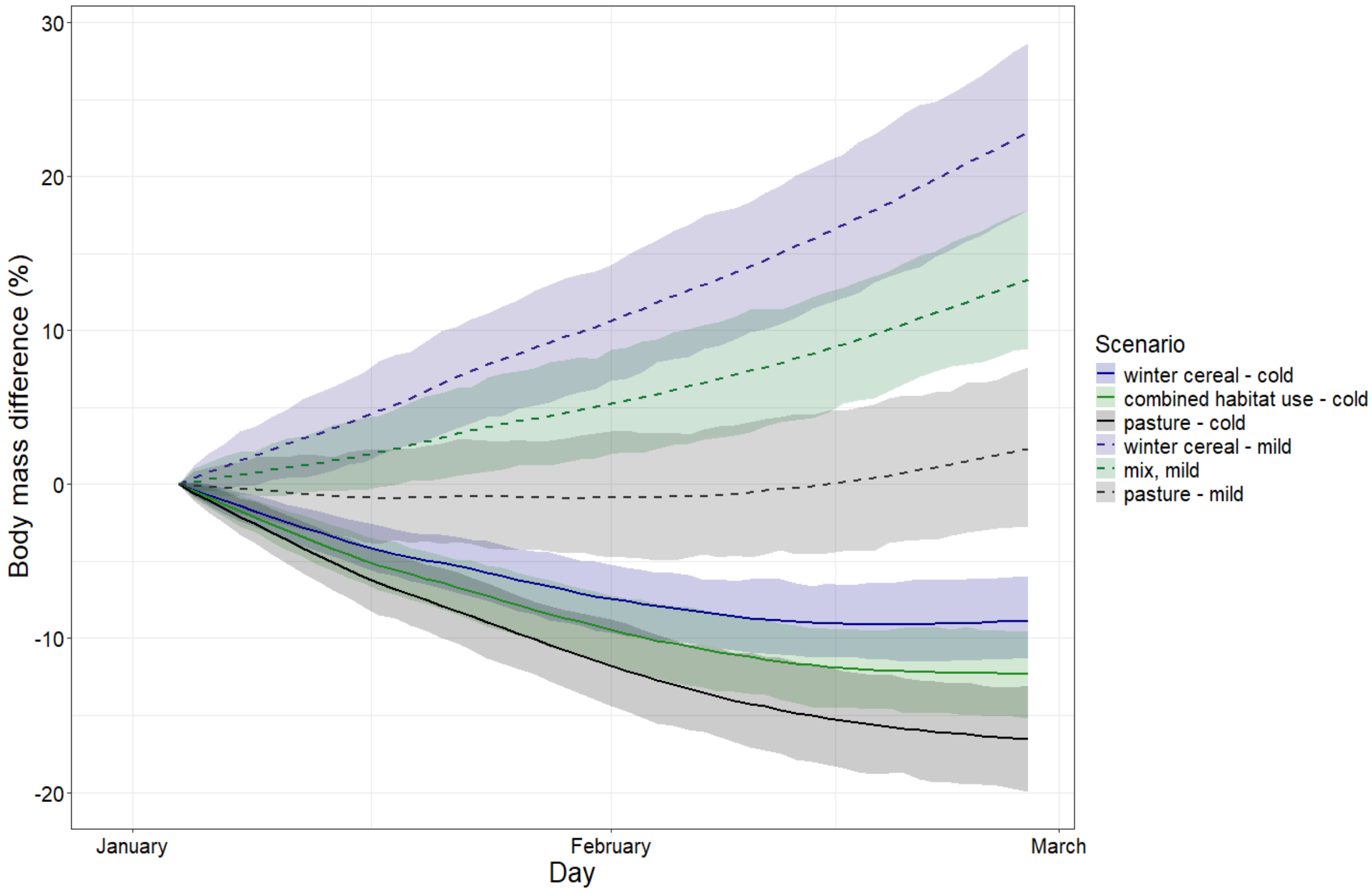

Fig. S3 Simulated relative body mass development shown as the relative difference for Taiga Bean Geese starting with an initial body mass of 3,487 g between 4 January and 28 February, including all six habitat and weather combinations. Solid lines represent the modelled body mass development under cold scenarios; the dashed lines represent the mild scenarios; the shaded areas indicate the 95% confidence intervals

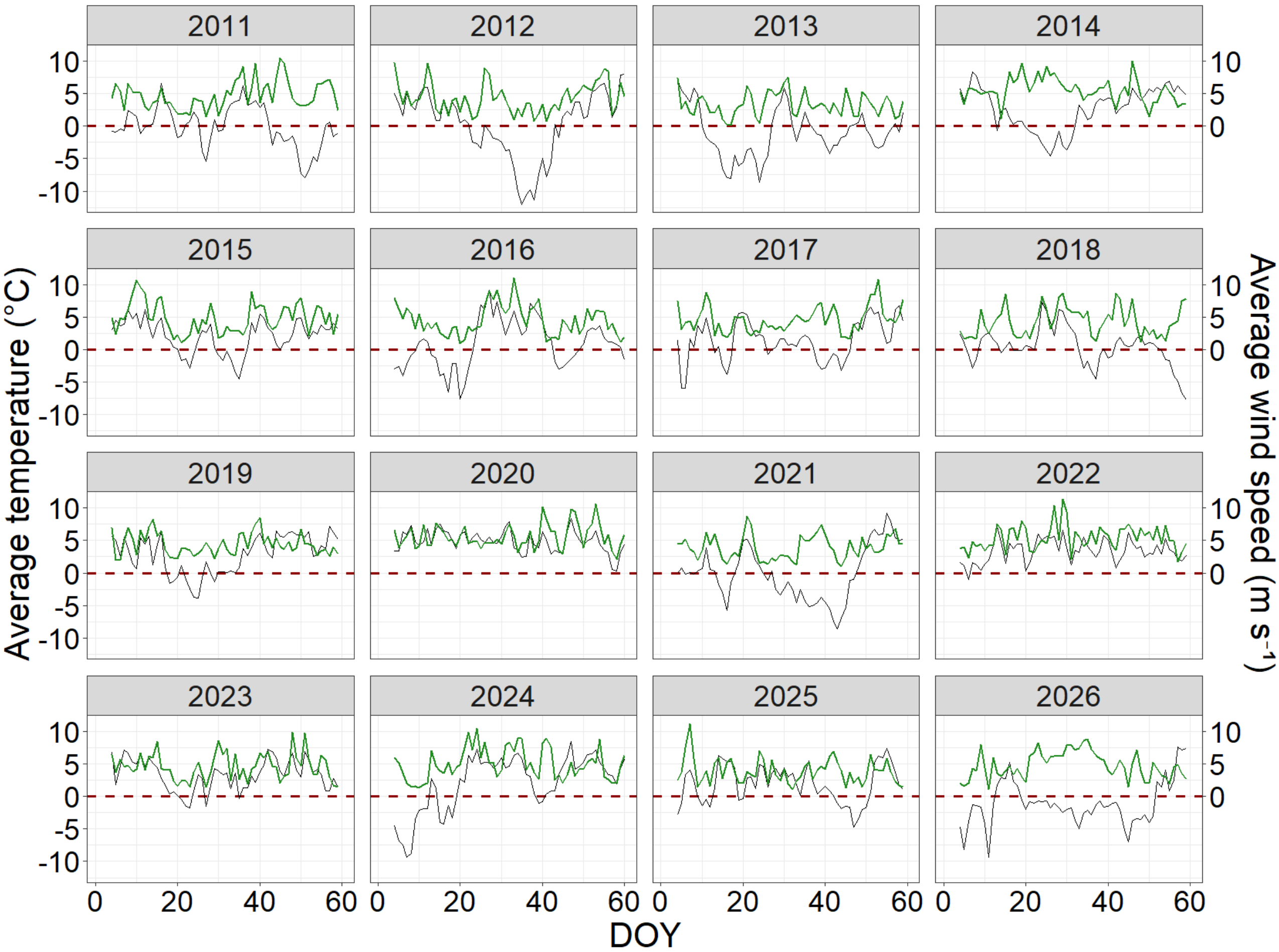

Fig. S4 Daily mean temperature (black) and wind conditions (green) per year in central Jutland, Denmark, from 4 January to 28 February between 2011 and 2026. The x-axis represents the day of the year (DOY), starting with 4 January. The red dashed line represents 0 °C, and 0 m/s. Data was derived from the Danish Meteorological Institute (DMI)

Supplementary Section S1: Habitat-specific activity proportions

Daily activity costs (kJ) were estimated for Taiga Bean Geese that spent the full day either in Nørreådalen, where birds primarily foraged on semi-natural pastures, or in Vinge, where foraging occurred mainly on winter cereals. Activity costs were calculated by multiplying the basal metabolic rate (BMR) of a mean winter body mass for Taiga Bean Geese (3,487 g) with the activity-based metabolic rate (AMR; Clausen et al. 2013).

AMR was calculated as:

$$AMR = \sum(behaviour\ proportion\ per\ day\ \times BMR\ multiplier)$$

To evaluate whether daily activity costs differed between areas or across time, we fitted a generalized linear mixed model with activity cost as the response variable, day and area as fixed effects, and individual as a random effect. Neither fixed effect was statistically significant (Tab. S3), indicating that estimated activity costs did not differ between areas or across the study period.

Given this result, and because the habitat scenarios described in the main text aimed to simulate local flight movements within Nørreådalen, we used the average behavioral probabilities derived from geese that remained in Nørreådalen throughout the day. Although behavioral proportions may differ between habitats (Clausen et al. 2013), non-flying behaviors resulted in similar activity-based metabolic rates for birds staying in Nørreådalen (1.56) and Vinge (1.51; Tab. S4), indicating that using the Nørreådalen behavioral probabilities did not substantially bias the activity cost calculations of geese simulated on winter cereals.

Table S3 Model estimates of the generalized linear mixed model

**Model**	**Estimate**	**SE**	**t**	**p**
glmer(activity costs ~ day + area + (1|nickname), family = Gamma				
day	0.106	0.336	0.314	0.754
area vinge	-23.454	37.783	-0.622	0.534

Table S4 Behavior proportions of Taiga Bean Geese that spent the full day in Nørreådalen vs. Vinge. Proportions were calculated for the entire day

**Behavior**	**Mean proportion Nørreådalen**	**SD**	**N**	**Mean proportion Vinge**	**SD**	**N**	**BMR multiplier**
Foraging	0.41	0.09	146	0.29	0.06	8	1.6
Preening	0.10	0.06	144	0.08	0.04	7	1.9
Resting	0.44	0.11	146	0.57	0.07	8	1.5
Sleeping	0.03	0.03	90	0.04	0.02	6	1.5
Flying	0.01	0.01	84	0.03	0.03	2	13.7

Supplementary Section S2: Energy intake daytime foraging:

Daily energy intake was estimated using habitat- and weather-specific energetic measurements reported by Vergin et al. (2025) (Table S5). In that study, fresh plant material and droppings of Taiga Bean Geese were collected from individuals foraging on winter cereals and semi-natural pastures under both cold and mild weather conditions. For each habitat–weather combination, dropping rates (*DR*) were estimated using the hourly block method. Plant and dropping samples were analyzed for ash content, acid detergent fiber (ADF; marker substance), gross energy content of plants (GE_p) and droppings (GE_d), and ash-free dry mass of droppings (M_f). Detailed descriptions of sampling procedures, laboratory analyses, and sampling conditions are provided in Vergin et al. (2025).

Net energy intake was calculated for the time spent on foraging grounds during daytime ($T_{foraging\ ground}$), which increased over the course of the winter season (see main text).

The excreted energy (E_e) was estimated as:

$$E_e = DR \times M_f \times GE_d$$

The ingested energy (E_i) was estimated as:

$$E_i = \frac{E_e}{1 - m}$$

Where metabolizability *m* was estimated as Dokter et al. (2018)

$$m = 1 - \frac{ADF_p}{ADF_d} . \frac{GE_d}{GE_p}$$

Finally, daytime net energy intake (I_{day}) was calculated as:

$$I_{day} = (E_i - E_e) \times \ T_{foraging\ ground}$$

Table S5 Energetic measurements of collected plant and feces samples (mean ± standard error) for Taiga Bean Geese, adapted after Vergin et al. (2025)

	Winter cereal		Pasture	
	Cold	Mild	Cold	Mild
Feces				
Defecation rate [n/hour]	11.34 ± 0.62	13.95 ± 5.37	14.49 ± 0.70	12.85 ± 0.71
Feces weight [g DW]	1.19 ± 0.03	1.83 ± 0.09	0.89 ± 0.03	1.00 ± 0.03
Organic matter [%]	73.14 ± 2.55	67.73 ± 2.09	87.06 ± 0.32	89.61 ± 0.63
ADF content [%]	28.51 ± 0.70	25.73 ± 0.72	30.28 ± 0.60	33.82 ± 0.82
Gross energy [kJ/g]	18.59 ± 0.16	17.44 ± 0.26	18.27 ± 0.04	18.42 ± 0.13
Plants				
Organic matter [%]	81.90 ± 4.36	75.38 ± 9.06	93.22 ± 0.57	92.94 ± 0.90
ADF content [%]	18.28 ± 1.08	17.90 ± 0.43	20.52 ± 1.85	21.52 ± 2.75
Gross energy [kJ/g]	21.76 ± 0.58	21.09 ± 0.34	20.21 ± 0.22	20.39 ± 0.22

Supplementary Section S3: Energy intake nocturnal foraging:

Nighttime energy intake was estimated by multiplying the duration of active nocturnal foraging with a per-minute energy intake rate (*IR*) derived from daytime foraging on semi-natural pastures. We assumed that intake rate did not differ between daytime and nocturnal foraging.

Daytime foraging activity was estimated as the proportion of time spent actively foraging on semi-natural pastures, based on positions recorded on foraging grounds during daylight hours. For each individual and day, this proportion was calculated and tested for temporal trends using a linear mixed-effects model with daily foraging proportion as the response, day as a fixed effect, and individual identity as a random effect. No significant temporal change was detected ($\beta = -0.002$, SE = 0.001, $t = -1.643$, $p = 0.103$); therefore, an average daily foraging proportion of 0.617 ± 0.133 SD over the winter period (January–February) was used.

The average foraging proportion was multiplied by the total time spent on the foraging grounds to obtain the active daytime foraging duration. Dividing the estimated daytime net energy intake (I_{day}) on semi-natural pastures by the active daytime-foraging duration (T_{f_day}) yielded the per-minute intake rate (*IR*):

$$IR = \frac{I_{day}}{T_{f\_day}}$$

Nighttime net energy intake (I_{night}) was then calculated by multiplying the nocturnal foraging duration (T_{f_night}) by this per-minute intake rate:

$$I_{night} = T_{f\_night} \times IR$$

References

Clausen KK, Clausen P, Fox AD, Fælled CC, Madsen J (2013) Varying energetic costs of Brent Geese along a continuum from aquatic to agricultural habitats: the importance of habitat-specific energy expenditure. J Ornithol 154:155-162. https://doi.org/10.1007/s10336-012-0881-8

Dokter AM, Fokkema W, Bekker SK, Bouten W, Ebbinge BS, Müskens G, Olff H, van der Jeugd HP, Nolet BA (2018) Body stores persist as fitness correlate in a long-distance migrant released from food constraints. Behav Ecol 29:1157-1166. https://doi.org/10.1093/beheco/ary080

Vergin L, Clausen KK, Madsen J (2025) The role of winter cereals as cold-weather refuge for Taiga Bean Geese wintering in Denmark. J Ornithol 166:803-814. https://doi.org/10.1007/s10336-025-02262-8
